# Supplementary material for: End Sequence Analysis Toolkit (ESAT) expands the extractable information from single-cell RNA-seq data
Source: Genome Res. 2016 Oct;26(10):1397–410. doi: 10.1101/gr.207902.116 (PMC5052061; doi:10.1101/gr.207902.116)
Supplement: Supplemental Material [file supp_gr.207902.116_Supp_Materials.tar.gz › ESAT-esat_v0.1/doc/html/classbroad_1_1core_1_1motif_1_1_sequence_motif_cluster.html]

ESAT: broad.core.motif.SequenceMotifCluster Class Reference


ESAT


- Main Page
- Related Pages
- Packages
- Classes
- Files

- Class List
- Class Index
- Class Hierarchy
- Class Members

All Classes Namespaces Files Functions Variables Enumerator Pages

- broad
- core
- motif
- SequenceMotifCluster

Public Member Functions |
Static Public Member Functions |
Static Public Attributes |
List of all members

broad.core.motif.SequenceMotifCluster Class Reference

Inheritance diagram for broad.core.motif.SequenceMotifCluster:

[legend]

Collaboration diagram for broad.core.motif.SequenceMotifCluster:

[legend]

|  |  |
| --- | --- |
| Public Member Functions | |
|  | SequenceMotifCluster (String corePattern, int minsuccessiveCores, int maxPatternDistance) throws SearchException |
|  | |
| List< SequenceRegion > | match (Sequence seq) |
|  | |
| List< SequenceRegion > | match (Sequence seq, boolean includeRegionSequence) |
|  | |
| Public Member Functions inherited from broad.core.motif.SequenceMotif | |
|  | SequenceMotif (Pattern motif) |
|  | |
|  | SequenceMotif (String corePattern, String identifier) throws SearchException |
|  | |
|  | SequenceMotif (String corePattern) throws SearchException |
|  | |
|  | SequenceMotif (String corePattern, int minsuccessive) throws SearchException |
|  | |
|  | SequenceMotif (String corePattern, int minsuccessive, String identifier) throws SearchException |
|  | |
| String | getId () |
|  | |
| String | getConsensusSequence () |
|  | |
| int | getNumPossibleKmers () |
|  | |
| Pattern | getMotif () |
|  | |
| List< SequenceRegion > | match (Sequence seq) |
|  | |
| boolean | equals (Object o) |
|  | |
| String | toString () |
|  | |
| int | hashCode () |
|  | |
| List< SequenceRegion > | match (Sequence geneSeq, List< SequenceRegion > list, int length) |
|  | |

|  |  |
| --- | --- |
| Static Public Member Functions | |
| static void | main (String[] args) throws NumberFormatException, SearchException, IOException |
|  | |
| Static Public Member Functions inherited from broad.core.motif.SequenceMotif | |
| static int | numPossibleKmers (String consensusSequence) |
|  | |
| static void | main (String[] args) throws NumberFormatException, SearchException, IOException |
|  | |

|  |  |
| --- | --- |
| Static Public Attributes | |
| static String | USAGE |
|  | |
| Static Public Attributes inherited from broad.core.motif.SequenceMotif | |
| static String | USAGE |
|  | |

|  |  |
| --- | --- |
| Additional Inherited Members | |
| Protected Member Functions inherited from broad.core.motif.SequenceMotif | |
| void | setMotif (Pattern motif) |
|  | |
| Pattern[] | getCoreMotifs () |
|  | |

## Constructor & Destructor Documentation

|  |  |  |  |
| --- | --- | --- | --- |
| broad.core.motif.SequenceMotifCluster.SequenceMotifCluster | ( | String | *corePattern*, |
|  |  | int | *minsuccessiveCores*, |
|  |  | int | *maxPatternDistance* |
|  | ) |  | throws SearchException |

Here is the caller graph for this function:

## Member Function Documentation

|  |  |  |  |  |  |
| --- | --- | --- | --- | --- | --- |
| static void broad.core.motif.SequenceMotifCluster.main | ( | String[] | *args* | ) | throws NumberFormatException, SearchException, IOException |

static

Parameters
:   |  |  |
    | --- | --- |
    | args |  |

Exceptions
:   |  |  |
    | --- | --- |
    | SearchException |  |
    | NumberFormatException |  |
    | IOException |  |

Here is the call graph for this function:

|  |  |  |  |  |  |
| --- | --- | --- | --- | --- | --- |
| List<SequenceRegion> broad.core.motif.SequenceMotifCluster.match | ( | Sequence | *seq* | ) |  |

|  |  |  |  |
| --- | --- | --- | --- |
| List<SequenceRegion> broad.core.motif.SequenceMotifCluster.match | ( | Sequence | *seq*, |
|  |  | boolean | *includeRegionSequence* |
|  | ) |  |  |

## Member Data Documentation

String broad.core.motif.SequenceMotifCluster.USAGE

static

**Initial value:**

= "Usage: SequenceMotifCluster TASK=<task\_num> <task\_args>\n" +

"\tTasks:\n" +

"\t\t1. Get list of motif matches: IN=<fasta sequence> MOTIF=<Core Motif e.g. YRNRY> SUSCCESSIVE=<number of consecutive core motifs> MINLENGTH=<minimum total length to report> MAXSPACING=<max spacing between motifs>\n"

---

The documentation for this class was generated from the following file:

- java/broad/core/motif/SequenceMotifCluster.java


---

Generated on Fri Aug 15 2014 15:10:54 for ESAT by  

 1.8.7
